# Prevalence of impaired renal function among childless men as compared to fathers: a population-based study

**DOI:** 10.1038/s41598-024-58479-9

**Published:** 2024-04-02

**Authors:** Michael Kitlinski, Aleksander Giwercman, Anders Christensson, Peter M. Nilsson, Angel Elenkov

**Affiliations:** 1 Department of Translational Medicine, Clinical Research Centre, Lund University, Malmö, Sweden; 2 Reproductive Medicine Centre, Skåne University Hospital, Malmö, Sweden; 3 Department of Nephrology, Skåne University Hospital, Malmö, Sweden; 4 Department of Clinical Sciences, Lunds University, Malmö, Sweden; 5 Faculty of Medicine, Medical University of Gdańsk, Gdańsk, Poland

**Keywords:** Kidney diseases, Disease prevention, Male factor infertility

## Abstract

Male reproductive impairment has been linked with an increased risk of numerous non-communicable diseases. Yet, epidemiological data on renal disease among subfertile men is scarce. Therefore, by using male childlessness as a proxy for male infertility, we aimed to investigate its association with renal function. Data was sourced from a population-based cohort including 22,444 men. After exclusion of men aged < 45 years (n = 10,842), the remaining men were divided into two groups: these being childless (n = 5494) and fathers (n = 6108). Logistic regression was applied to explore the association between male childlessness and renal impairment. Childless men as compared to fathers, were more likely to have an estimated-glomerular filtration rate < 60 ml/min/1.73m^2^ (OR 1.36, 95 CI 1.08–1.70; p = 0.008). After adjustment for age, marital status, smoking habits, diabetes, hypertension and other components of metabolic syndrome, childless men were also more likely to have dipstick proteinuria (OR 1.85, 95 CI 1.16–2.95; p = 0.01). With the growing panorama of disease associated with male reproductive impairment, men with fertility issues may constitute a target population with potential benefit from closer follow-up of their renal function.

## Introduction

Mounting evidence has shown associations between impaired male reproductive function and non-communicable diseases. Health insurance-based databases have found infertile men to be at an increased risk of being diagnosed with ischemic heart disease and diabetes^1,2^. Male childlessness, being an infertility proxy, is associated with hyperlipidemia, hyperglycemia, hypertension, and elevated risk of cardiovascular-related mortality^3^. Fathers who conceived children using intracytoplasmic sperm injection, an indicator of severely impaired semen quality, were reported to be more likely to be prescribed medications for the metabolic syndrome and hypertension^4^. Metabolic and cardiovascular disturbances described above have also been associated with the development of chronic kidney disease (CKD)^5^. The direct relationship between male infertility and renal disease however remains largely unexplored. Limited evidence for an association between both conditions comes from a single report from Eisenberg et al. showing that infertile men are at increased risk of developing renal disease^2^. A limitation of this study was lack of adjustment for other comorbidities strongly linked to renal dysfunction, such as hypertension and diabetes.

Measurement of creatinine-based estimated glomerular filtration rate (eGFR) and dipstick test for protein in urine are routinely used for screening and evaluation of renal function. Markers of potential kidney damage, such as a single measurement of dipstick proteinuria or eGFR < 60 mL/min/1.73 m^2^ are associated with 3–15 fold, respectively, 2–threefold, increased risk of developing end-stage renal disease^6^. Thus, to avoid the comorbid burden of the later stages of CKD, identification of patients at an early stage of renal impairment, which often is asymptotic, remains essential.

The Malmö preventive project (MPP) is a longitudinal, population-based study established in the 1970s^7^, containing data on creatinine levels, urine dipstick results, as well as information on the participants´ fatherhood status. The aim of this study was, therefore, to investigate if male childlessness (being a proxy for male subfertility) is linked to impaired renal function defined as eGFR < 60 mL/min/1.73 m^2^ and/or presence of dipstick proteinuria.

## Results

### Study population

Among the 11,602 men, aged 45 or older, 5494 (47.3%) were childless and 6108 (52.7%) were fathers, respectively. Mean (SD) age in childless men was 49.4 (4.49) years compared to 48.1 (3.26) years among fathers. Fathers were also more commonly married (89.2%) when compared to childless men (67.3%). Fathers were also less commonly obese (5.4% vs 9.5%), hyperlipidemic (27.5% vs 32.7%) or hypertensive (53.4% vs 57.8%), when compared to childless men. Further baseline characteristics on these men can be found in Table 1.

**Table 1:** Baseline characteristics of childless men and fathers included in the cohort.

	Childless men (n = 5494)	Fathers (n = 6108)
Age mean (SD), years	49.4 (4.49)	48.1 (3.26)
eGFR mL/min/1.73m^2^		
> 90	1805 (32.9%)	1705 (27.9%)
89–60	3397 (61.8%)	4096 (67.1%)
59–45	123 (2.24%)	102 (1.67%)
44–30	11 (< 1%)	7 (< 1%)
29–15	3 (< 1%)	1 (< 1%)
< 15	3 (< 1%)	2 (< 1%)
Dipstick proteinuria results		
Negative	5000 (91.0%)	5729 (93.8%)
Trace	332 (6.1%)	266 (4.3%)
1 +	44 (< 1%)	29 (< 1%)
2 +	16 (< 1%)	2 (< 1%)
Marital status		
Married	3693 (67.2%)	5446 (89.2%)
Unmarried, divorced or widower	1772 (32.3%)	655 (10.7%)
Smoker		
Yes	3223 (58.7%)	2581 (42.3%)
No	2271 (41.3%)	3527 (57.7%)
Comorbidities		
Hypertension	3171 (57.8%)	3261 (53.4%)
Impaired glucose sensitivity	1192 (21.7%)	1417 (23.2%)
Obesity	521 (9.5%)	330 (5.4%)
Hyperlipidemia	1794 (32.7%)	1679 (27.5%)

Percentages do not add up to 100% in all parameters due to missing data.

### Association between markers of renal disease and fatherhood status

In all, 3.1% of childless men had an eGFR < 60 mL/min/1.73 m^2^, compared to the 2.3% in fathers. In the logistic regression analysis, childless men, as compared to fathers, were more likely to present with eGFR < 60 mL/min/1.73 m^2^ (OR 1.36, 95 CI 1.08–1.70; p = 0.008; Table 2). The association remained statistically significant after adjustment for socioeconomic factors (aOR 1.54, 95 CI 1.20–1.97; p < 0.001; Table 2). However, In the 2nd adjusted model, the association was no longer statistically significant (aOR 1.13, 95 CI 0.88–1.45; p = 0.34; Table 2).

**Table 2:** Likelihood of dipstick proteinuria and eGFR < 60 ml/min/1.73m^2^ at baseline screening in childless men, when compared to fathers.

	Unadjusted model		Adjusted model 1		Adjusted model 2	
	Odds ratio (95% CI)	P-value	Odds ratio (95% CI)	P-value	Odds ratio (95% CI)	P-value
Dipstick proteinuria	2.18 (1.41–3.36)	< 0.001	1.80 (1.10–2.97)	0.02	1.85 (1.16–2.95)	0.01
eGFR < 60 ml/min/1.73m^2^	1.36 (1.08–1.70)	0.008	1.54 (1.20–1.97)	< 0.001	1.13 (0.88–1.45)	0.34
eGFR < 60 ml/min/1.73m^2^ with dipstick proteinuria	6.35 (1.86–21.7)	0.003	6.03 (1.69–21.5)	0.006	6.10 (1.71–21.7)	0.005

Covariates in adjusted model 1: marital status (married vs unmarried, divorced or widower) and social class (non-manual employee [high, medium, or low level], manual employee [skilled or unskilled], or self-employed).

Covariates in adjusted model 2: age, marital status (married vs unmarried, divorced or widower), smoking status (yes/no), hypertension, impaired glucose sensitivity, obesity and hyperlipidemia.

Dipstick proteinuria defined as + 1 or + 2 dipstick result.

Childless men were also more likely to have dipstick proteinuria at baseline (7.1% in childless men and 4.9% in fathers, OR 2.18, 95 CI 1.41–3.36; p < 0.001). Even after adjustment for all covariates in the 2nd adjusted model, as compared to fathers, childless men had a statistically significant higher likelihood of having dipstick proteinuria (aOR 1.85, 95 CI 1.16–2.95; p = 0.01; Table 2). Childless men were at higher odds of having eGFR < 60 mL/min/1.73 m^2^ combined with dipstick proteinuria (0.3% vs 0.05%; OR 6.35, 95 CI 1.86–21.7; p = 0.003; Table 2). This association was still statistically significant in both adjusted models (Table 2).

In sensitivity analysis where men with CKD stage 4 and 5 were excluded, a higher likelihood was still present in the unadjusted model for eGFR < 60 mL/min/1.73 m^2^ among childless men when compared to fathers (OR 1.34, 95 CI 1.07–1.69; p = 0.012). Similarly, male childlessness was associated with an increased likelihood of dipstick proteinuria (OR 2.07, 95 CI 1.33–3.21; p = 0.001). After adjustment for age, marital status, and comorbidities linked to CKD, childless men in sensitivity analysis were still more likely to have eGFR < 60 mL/min/1.73 m^2^ together with dipstick proteinuria (aOR 4.95, 95 CI 1.35–18.1; p = 0.016), as compared to fathers.

## Discussion

In this population-based study we found that childless men, as compared to fathers, are more prone to show signs of renal disease as decreased eGFR and dipstick proteinuria. The likelihood of dipstick proteinuria, with or without concomitant decrease in eGFR, remained statistically significant even after adjustment for comorbidities and traits known to be linked to impaired renal function.

Research data throwing light on the association between renal disease and male infertility, are rather scarce. Using insurance claims data, Eisenberg et al. found men with an infertility diagnosis to be at a 1.6-fold increased risk of developing renal disease, with the risk-estimates being the highest in those with azoospermia^2^ – the most severe form of male reproductive impairment. The study did, however, not address the issue of type and severity of the over-represented renal pathologies and did not adjust for the leading causes of CKD in western countries, i.e. diabetes and hypertension. Interestingly, in our study, after adjustment for these comorbidities as well as for other components of the metabolic syndrome, a statistically significant higher likelihood of dipstick proteinuria, and decreased eGFR together with dipstick proteinuria, respectively, was still present. This could suggest a direct association between male childlessness and impaired renal function, not mediated via traditional cardiovascular and/or metabolic disturbances. It has been suggested that a significant proportion of impaired male fertility are linked to adverse environment and/or lifestyle related exposures in early fetal life^8^. It appears plausible, but impossible to prove in the current study set up, that such pathogenetic factors might also be responsible for development of renal disease.

Our study also found childless men to be at a twofold higher likelihood of having dipstick proteinuria, when compared to fathers. Accumulated data from nationwide population-based studies suggests that low-grade albuminuria is associated with a higher cancer-related mortality and an increased risk of cardiovascular diseases^9,10^. There is also an association between low eGFR and an increased cancer incidence^11^. As it has been shown that men with a decreased fertility potential are at an increased risk of developing the above-mentioned diseases^2,12^, the higher prevalence of dipstick proteinuria and decreased eGFR in those men could etiologically and/or pathogenetically be linked to these increased risk-estimates.

For infertile couples seeking fertility care, conventional semen sample analysis, as recommended by the *World Health Organization*, remains the staple for male fertility evaluation^13^. Over the last couple of years, a discussion has emerged to extend the infertility investigation by also including a clinical, endocrinological and in certain cases genetic investigations in routine work-up of subfertile men^14,15^. Using solely basic semen parameters to evaluate the etiology of male reproductive impairment is insufficient as unexplained infertility is present in up to 30% of infertile couples after routine evaluation^16^. Moreover, as previously mentioned, multiple studies have shown male reproductive impairment to have negative long-term health consequences, such as increasing the risk of metabolic, cardiovascular diseases, and certain malignancies^2,3,12,17,18^. As men seeking fertility evaluation, access the healthcare system at a relatively young age, this is sometimes the first, if not the only, chance for such men to undergo basal health examinations. Presence of proteinuria is usually an early manifestation of kidney impairment occurring before the evident decline in eGFR. Thus, diagnosing this condition by use of a simple test, might contribute to prevention of serious renal issues. Our finding that childless men were more likely to have dipstick proteinuria, with or without an eGFR < 60 mL/min/1.73 m^2^, as compared to fathers, supports the idea of implementing additional clinical measures in the population of sub-fertile men. Considering that a single measurement of dipstick proteinuria increases the 25-year risk of developing ESRD^6^, childless and infertile men may represent a high-risk group which would potentially benefit from having their renal status monitored simultaneously as undergoing fertility evaluation, something which is not currently recommended.

A major strength of our study is use of a large population-based cohort containing 10,000 men with robust data concerning medical history and laboratory values. Access to complete data on the baseline profile, allowed us to obtain risk-estimates for renal dysfunction adjusted for cardiovascular disease, diabetes, and other causes of CKD. There are, however, several limitations that need to be addressed. Firstly the major concern is that almost 50% of men 45 or older were categorized as being childless uring in 1980s this proportion in Sweden being reported to be in Sweden, close to 25%^19^. We have relied on high quality registry data, in order to classify men into fathers or childless. Although we do not have full explanation for the high percentage seen in our cohort, it could at least partially be attributed to missing fatherhood data in the STAS register before 2005. Although reporting data to the STAS has been mandatory by law, an underreporting of adopted children or those following conception with donated sperms might have occurred. The same is true for men who have achieved fatherhood outside of Sweden before or after baseline examination. However, the events described above cannot fully explain the high proportion of childless men in our study cohort. Although any misclassification leading to some fathers being categorized as childless would probably lead to bias toward the null hypothesis, our results need to be interpreted with caution.

Secondly, male childlessness is not the most robust surrogate for impaired spermatogenesis because it could be related to rather social than male biological determinants, as well as to partner´s infertility. However, we tried taking this into account by adjusting for marital and social status – parameters related to fertility potential, awareness and social aspects influencing family planning^20^. Nonetheless, even with such fine adjustments, presence of voluntarily childless men still could have influenced our results, but such a misclassification would also rather have underestimated than overestimated the associations identified in this study. The dipstick proteinuria parameter was evaluated by visual reading which affects the precision of the results. To minimize this, we divided the results into two groups: non-proteinuric and proteinuric. It is important to mention that measuring serum creatinine using the Jaffe method has its limitations. Although measuring renal function with cystatin C-based equations remains superior over creatinine-based estimations, this should not cause alterations in the results when it comes to comparing renal function between childless men and fathers. Though, further research on renal function among infertile men is required, preferably with more accurate renal measures such as urinary albumin-creatinine ratio, microalbuminuria, and cystatin C-based eGFR, as dipstick proteinuria assessment has low specificity and sensitivity for early kidney impairment.

In conclusion, independent of socioeconomic status, age, or traditional risk factors such as hypertension, metabolic syndrome and diabetes, childless men as compared to fathers, were at a higher likelihood of presenting with dipstick proteinuria, with or without concomitantly decreased eGFR. Men presenting with fertility issues might represent a target population which would benefit from having their kidney function investigated.

## Methods

### Subjects

We retrieved data from the Malmö Preventive Project—a population-based prospective cohort study including 22,444 men from the general population (representing 71% of all invited), enrolled between the years 1974–1994 aged 25 to 63 years at baseline. All participants filled out a baseline questionnaire regarding lifestyle habits, familial and medical history. They also provided a urine sample and underwent baseline physical examination and laboratory tests providing data on parameters such as body mass index (BMI; kg/m^2^), blood pressure (mmHg), serum creatinine (µmol/L), fasting blood glucose and triglycerides levels (mmol/l). Since some men could have been too young at baseline for achieving their final fatherhood status, in the presented study those younger than 45 years at baseline were excluded (n = 10,842). This subsequently resulted in the inclusion of 11,602 men aged ≥ 45 years. Thereafter, using unique personal identification numbers, data on the number of children at baseline were sourced from the Swedish Tax Agency Statistics (STAS), allowing for grouping of the included men based on the fatherhood status. Men who had no children registered in the STAS, or who fathered a child after baseline enrolment (n = 583), were considered as childless, while those with ≥ 1 child born before or at baseline enrolment were classified as fathers. Information concerning marital status (married, unmarried, divorced or widower) and social class which is based on occupation (non-manual employee [high, medium, or low level], manual employee [skilled or unskilled], or self-employed) was also derived from the STAS. More detailed description of the socioeconomic variables has been previously published^20^.

### Assessment of renal function and proteinuria

Serum creatinine was measured using the Jaffe’s alkaline picrate method. eGFR was calculated using the 2021 CKD-EPI creatinine formula^21^. A semi quantitative urine dipstick test (Rediatest, Boehringer Mannheim GmbH, Mannheim, Germany) at baseline enrolment was used to evaluate the presence of proteinuria. Dipstick results were categorized into negative, trace, 1 + , and 2 + proteinuria. 1 + or 2 + dipstick result was defined as positive dipstick proteinuria, while negative or trace was not.

### Ethical permission

Ethical approval for this study was given by the Ethics committee at Lund University (LU 85/2004): The participants signed a written informed consent. Research on all research participants was performed in accordance with the Declaration of Helsinki. This study was performed in accordance with all relevant guidelines/regulations.

### Statistical analysis

Associations between male childlessness, eGFR < 60 mL/min/1.73 m^2^ and/or dipstick proteinuria was evaluated using logistic regression models, providing crude odds ratio (OR) and adjusted odds ratio (aOR) with a 95% confidence interval (95% CI). Two adjusted models with the following covariates were used:

1. Adjustments in this model took into consideration men’s marital, socioeconomic and occupational status, which are linked to fertility potential and number of children conceived^22^. Covariates in the first adjusted model were marital status (married vs unmarried, divorced or widower) and social class (non-manual employee [high, medium, or low level], manual employee [skilled or unskilled], or self-employed).
2. This model adjusted for age (continuous), marital status (married vs unmarried, divorced or widower), smoking status (yes/no) and comorbidities which have been linked to an increased risk of CKD, those being hypertension (defined as systolic blood pressure > 140 mmHg and/or diastolic blood pressure > 90, after laying down for 10 min, or intake of antihypertensive medications), impaired glucose sensitivity (defined as fasting blood glucose ≥ 5.6 mmol/L, or post-load glucose ≥ 11 mmol/L at 120 min after glucose tolerance test, or reported history of diabetes/intake of ACT category of A10 medications, or at least two HbA_1c_-values ≥ 6.0% [at separate dates] in the HbA_1c_-register at the Department of Clinical Chemistry, Malmö), obesity (defined as body mass index > 30 kg/m^2^), and hyperlipidemia (defined as fasting blood triglycerides > 1.7 mmol/L).

Severely decreased semen quality might be a consequence of severe CKD^23^. Thus, a sensitivity analysis, excluding men with CKD stage 4 and 5 (eGFR < 30 mL/min/1.73 m^2^) was also performed.

A p-value < 0.05 was considered as statistically significant. All statistical analyses were conducted using IBM SPSS Statistics version 28 (IBM Corp.).

## Data Availability

Scientists can obtain anonymized data by contacting the corresponding author, if the required conditions are met under the Swedish law.
